# Study on expression of CDH4 in lung cancer

**DOI:** 10.1186/s12957-016-1083-2

**Published:** 2017-01-17

**Authors:** Zhupeng Li, Dan Su, Lisha Ying, Guangmao Yu, Weimin Mao

**Affiliations:** 1 Department of Cardiothoracic Surgery, Shaoxing People’s Hospital, 568 Zhongxin Road, Shaoxing, Zhejiang China; 2 Cancer Research Institute, Zhejiang Cancer Hospital, 38 Guangji Road, Hangzhou, Zhejiang China; 3 Department of Thoracic Surgery, Zhejiang Cancer Hospital, 38 Guangji Road, Hangzhou, Zhejiang 310022 China

**Keywords:** CDH4, Lung cancer, Tumor suppressor gene

## Abstract

**Background:**

The human CDH4 gene, which encodes the R-cadherin protein, has an important role in cell migration and cell adhesion, sorting, tissue morphogenesis, and tumor genesis. This study analyzed the relationship of CDH4 mRNA expression with lung cancer.

**Methods:**

Real time PCR was applied to detect CDH4 mRNA transcription in 142 paired cases of lung cancer and noncancerous regions.

**Results:**

No correlation was identified between CDH4 mRNA expression and gender, age, lymphnode metastasis, TNM stage, family history, smoking state, drinking state (*P* > 0.05), but grade and histotype (*P* < 0.05). The relative CDH4 mRNA value was remarkably decreased in lung cancer tissues compared with noncancerous tissues (*P* = 0.001).

**Conclusions:**

We found that CDH4 mRNA expression was associated with grade and histotype. What is more, the relative CDH4 mRNA value was decreased in the lung cancer tissues. Our results suggested that CDH4 might be a putative tumor suppressor gene (TSG) in lung cancer.

## Background

Lung cancer has the highest morbidity and mortality in malignant tumors and with a total of 1.5 million deaths annually worldwide [1]. In the patients with lung cancer, though diagnosis and treatment have been greatly advanced, the 5-year survival rate remains low [2, 3]. Many etiological factors correlate with lung cancer, for example, genetic susceptibility, environmental factors, smoking, and so on, but the mechanism of lung cancer development and progression is yet unclear.

Cadherins are transmembrane adhesion, calcium-dependent molecules, which have a lot of functions, for instance, cell signaling, cell–cell cohesion, and inhibition of apoptosis [4]. Cadherins regulate cell growth, mobility, and differentiation by binding through the cytoplasmic domain to b-catenin or c-catenin [5]. More than 100 cadherins have been found with diverse protein structures.

R-cadherin is a classic cadherin, which is important for the differentiation of kidney, striated muscle, brain, and so on [6, 7]. R-cadherin plays a critical role in maintaining cell polarity and tissue architecture in normal gastrointestinal epithelial tissue [8].

The human CDH4 gene encodes the R-cadherin protein, which is located on chromosome 20q13.3 [9]. Previous reports have showed that R-cadherin is involved in cell migration, cell adhesion, sorting, tissue morphogenesis, and tumorgenesis. CDH4 might be a tumor suppressor gene in various cancers, such as nasopharyngeal carcinoma, gastric cancer, and colorectal cancer [10–12]. However, the CDH4 expression in lung cancer and its’ correlation with lung cancer clinical characteristics were still unclear.

In this study, we first investigated the transcription levels of CDH4 mRNA in lung cancer tissues and paired noncancerous regions, to explore the relationship between CDH4 mRNA and clinical characteristics, and attempted to illustrate that CDH4 was a tumor suppressor gene (TSG) in lung cancer.

## Methods

### Clinical tissue samples

We collected 142 pair cases of fresh carcinoma and normal tissues from patients with lung cancer, all of them underwent surgery in Zhejiang Cancer Hospital from May 2012 to May 2013. Normal tissues were obtained as far (at least 5 cm) from the tumor tissue as possible. We froze the samples in liquid nitrogen and stored them at −80 °C as soon as possible. All of the patients did not receive preoperative radiochemotherapy. Patient clinical characteristics are summarized in Table 1. The classification criteria of International Association for the Study of Lung Cancer and the World Health Organization (IASLC/WHO) were used for histodiagnosis [13]. The TNM stage of the disease was determined by the new IASLC staging methods [14]. Our research was approved by Zhejiang Cancer Hospital Ethics Committee. Informed consents were provided for all patients before surgery.

**Table 1:** The correlation between clinical characteristics and CDH4 mRNA expression in lung cancer tissue

Variable	*N*	CDH4 mRNA	*P*
		Median (mean, range)	
Gender			0.137
Female	28	0.088(0.190, 0.019–1.367)	
Male	114	0.075(0.141, 0.012–2.661)	
Age			0.539
<65	99	0.080(0.167, 0.013–2.661)	
≥65	43	0.074(0.115, 0.012–0.517)	
Grade			0.013*
“High”–“middle”	71	0.068(0.119,0.012-0.932)	
“Middle-low”–“low”	63	0.108(0.197,0.013-2.661)	
Censoring	8	0.053(0.071,0.022-0.175)	
Histotype			0.001*
Squamous	77	0.067(0.116, 0.012–1.367)	
Adenocarcinoma	58	0.125(0.203, 0.013–2.661)	
Others	3	0.084(0.096, 0.081–0.125)	
SCLC	4	0.108(0.106,0.069-0.139)	
Lymph node metastasis			0.733
0	69	0.092(0.161, 0.012–1.367)	
1	21	0.067(0.113, 0.013–0.408)	
2–3	26	0.073(0.187, 0.016–2.661)	
4–24	24	0.086(0.120, 0.020–0.517)	
Censoring	2	0.100(0.100, 0.078–0.123)	
Stage			0.580
I–II	96	0.082(0.172, 0.012–2.661)	
III–IV	46	0.079(0.106, 0.020–0.517)	
Family history			0.404
Yes	25	0.070(0.115, 0.012–0.666)	
No	105	0.083(0.167, 0.013–2.661)	
Censoring	12	0.070(0.085, 0.040–0.172)	
Smoking state			0.476
Yes	100	0.083(0.149,0.012-2.661)	
No	31	0.080(0.180, 0.019–1.367)	
Censoring	11	0.072(0.088, 0.040–0.172)	
Drinking state			0.216
Yes	73	0.068(0.164, 0.013–2.661)	
No	58	0.090(0.146, 0.012–1.367)	
Censoring	11	0.072(0.088, 0.040–0.172)	

*A *p* value ≤0.05 was considered statistically significant

### Reverse transcription PCR

We extracted total RNA in tissues with Trizol kit ((Invitrogen, USA). The quality and concentration of RNA were evaluated by spectrophotometer. RT-PCR was conducted with One Step SYBR® Ex Taq™ qRT-PCR Kit (Takara, Japanese). The RT-PCR reaction was conducted in a 25-μl volume, containing TaKaRa Ex Taq HS (5 U/μl), 0.5 μl; PCR Forward Primer (10 μM), 0.5 μl; RTase Enzyme Mix, 0.5 μl; total RNA, 2 μl; PCR Reverse Primer (10 μM), 0.5 μl; RNase Free dH_2_O, 8.5 μl; 2 × One Step SYBR® RT-PCR Buffer, 12.5 μl. Amplification reactions were carried out as follows: stage 1: reverse transcription hold: 42 °C for 5 min, 95 °C for 10 s; stage 2: PCR reaction repeat: 40 cycles of 95 °C for 5 s, 60 °C for 20 s; stage 3: melt curve. The amplification reactions of the GAPDH were performed in the same tube. The GenBank sequences were used to design CDH4 and GAPDH primer sets. CDH4 reverse primer, 5′-GAAGACCAGCAGGGAGTCATAG-3′, CDH4 forward primer, 5′-CACCAAAAACAACGTCTACGAG-3′; GAPDH reverse primer, 5′-GAAGATGGTGATGGGATTTC-3′, GAPDH forward primer, 5′-GAAGGTGAAGGTCGGAGTC′. The ABI step-one plus performed the quantitative real-time PCR testing. Ct value was calculated with SDS2.1 software. GAPDH was used as a reference gene, when detected the relative expression of CDH4 mRNA. We used the 2^−△△Ct^ method to calculate the relative quantity (RQ) value.

### Statistical analysis

The obtained results were not normal distribution value (*P* < 0.05), analyzed by the Komlogorov-Smirnov test. So, analyses of the CDH4 mRNA relative value between the different groups were carried out by the Kruskall–Wallis test. The correlation between relative CDH4 mRNA value and clinical characteristics was estimated with the Spearman test. SPSS17.0 software was used for all statistical analyses. Bilateral test *P* < 0.05 was considered to have statistical significance.

## Results

### Correlation of CDH4 mRNA expression with clinical parameters of lung cancer patients

No correlation was identified between CDH4 mRNA expression and gender, age, lymphnode metastasis, TNM stage, family history, smoking state, drinking state (*P* > 0.05), but grade and histotype (*P* < 0.05) (Table 1). CDH4 mRNA expressed higher in “middle-low”–“low” grade compared with “high”–“middle” grade. In adenocarcinoma, SCLC, other histotypes, and squamous lung cancer, the CDH4 mRNA expression was dropped off.

### CDH4 mRNA expression in lung cancer tissues and normal tissues

CDH4 mRNA relative value was widely distributed among these tested patients. In the group of tumor tissues, the CDH4 mRNA relative value ranged between 0.01 and 2.66 (median 0.08), whereas in the groups of normal tissues, it ranged between 0.03 and 3.01 (median 0.26). The relative value of CDH4 mRNA in lung cancer tumor tissues (mean 0.15) was lower than those of normal tissues (mean 0.32, *P* < 0.05) (Table 2).

**Table 2:** The nonparametric tests of CDH4 mRNA expression in lung tumor tissues

	Median (mean, range)	*P* value
Normal	0.26(0.32, 0.03–3.01)	<*0.001*
Cancer	0.08(0.15, 0.01–2.66)	

## Discussion

In our research, we first conducted RT-PCR examining CDH4 mRNA expression in 142 paired cases of lung cancer tissues and noncancerous tissues. We found that CDH4 mRNA expression was not associated with gender, age, lymph node metastasis, TNM stage, family history, smoking state, drinking state, but histotype and grade. CDH4 mRNA was expressed higher in “middle-low”–“low” grade compared with “high”–“middle” grade. In adenocarcinoma, SCLC, other histotypes, and squamous lung cancer, the CDH4 mRNA expression was dropped off. It suggested that CDH4 might play an important role in tissue differentiation. This was consisted with the precious studies [15–17]. In Duguay et al.s’ experiment, the expression levels of cadherins were different in fibroblasts. It suggested that we could sort cell by different levels of cadherins cell surface expression as well as subtype [15]. What is the most, we found that CDH4 mRNA expression was significantly downregulated in lung tumors compared with normal tissues. In some cancers, for example, leiomyoma and ovarian carcinoma, CDH4 was treated as proto-oncogene, CDH4 gene expression was upregulated [18]. However, in some other cancers, such as nasopharyngeal carcinoma, gastric cancer, and colorectal cancer, CDH4 acted as anti-oncogene, CDH4 gene expression was downregulated[11, 12]. In lung cancer, our study suggested that CDH4 might serve as tumor surpressor gene, though more research need to be conducted.

## Conclusions

In conclusion, our study first reported that the expression levels of CDH4 mRNA were decreased in the lung cancer tissues. CDH4 might be a tumor suppressor gene in lung cancer. But further studies will be necessary to confirm this result.

## Abbreviations

CDH4: Cadherin 4
GAPDH: Reduced glyceraldehyde-phosphate dehydrogenase
IASLC: International Association for the Study of Lung Cancer
RT-PCR: Reverse transcription-polymerase chain reaction
TSG: Tumor suppressor gene
